# Malaria infection among children under-five: the use of large-scale interventions in Ghana

**DOI:** 10.1186/s12889-018-5428-3

**Published:** 2018-04-23

**Authors:** Clifford Afoakwah, Xin Deng, Ilke Onur

**Affiliations:** School of Commerce, UniSA Business School, University of South Australia, GPO BOX 2471, Adelaide, SA 5001 Australia

**Keywords:** IRS, ITN use, Malaria infection, Children under-five, Sub-Saharan Africa

## Abstract

**Background:**

Despite the significant investments to control malaria infection rates over the past years, infection rates remain significant in sub-Saharan Africa. This study investigates the association with use of large-scale malaria interventions such as: Indoor Residual Spraying (IRS), Insecticide Treated bed-Nets (ITN), and Behaviour Change Communication (BCC) strategies, and the prevalence of malaria among children under-five in Ghana.

**Methods:**

Cross-sectional data on 2, 449 children aged 6 to 59 months who were tested for malaria, through Rapid Diagnostic Test (RDT), are drawn from the recent wave of the Ghana Demographic and Health Surveys (GDHS 2014). We use a logit model to analyse the heterogeneous association between control measures and malaria infection among under five children of different age cohorts and household poverty statuses.

**Results:**

Our estimates suggest that IRS offers much more protection than ITN use. The odds of malaria infection among children who sleep in IRS is significantly lower (odds ratio [OR] = 0.312; 95% CI -1.47 -0.81; *p* = 0.00) compared to those who are not protected. This association is even high (odds ratio [OR] = 0.372; 95% CI -1.76 -1.02; *p* = 0.00) among children in poor households protected by IRS compared to those who have no IRS protection. ITN use did not have a significant association with malaria infection among children, except among children whose mothers have at least secondary education. For such children, the odds of malaria infection are significantly lower ([OR] =0.545; 95% CI = − 0.84 -0.11; *p* = 0.011) compared to those who are not protected. Regarding BCC strategies, we found that malaria education through television is the best strategy to covey malaria education as it significantly reduces the odds of malaria infection ([OR] =0.715; 95% CI = − 0.55 -0.10; *p* = 0.005) compared to those who do not received malaria education via television. BCC strategy via print media has a significant but limited protection for children of educated mothers.

**Conclusion:**

Policy makers should direct more resources to IRS, especially in communities where the use of ITN is less likely to be effective, such as poor and rural households. The distribution of ITNs needs to be accompanied with education programs to ensure its best protection.

## Background

Malaria is a life-threatening disease caused by infection through the bite of a female *anopheles* mosquito and poses the biggest health threat to children under five and pregnant women in malaria-prone areas such as sub-Saharan Africa. It is considered an endemic disease and a public health problem in Ghana due to the significant death toll associated with it [1]. It is also leads to poverty and low productivity due to human development, as well as due to its financial burden on households and the economy [2, 3]. In Ghana it is estimated that every malaria episode recorded corresponds to an average of 5 workdays lost; 3 days to the patient and 2 days to the caretaker [4]. Figures from the World Health Organisation (WHO) [5] also suggest that malaria alone accounts for about 4 sick days in a month, and 6.4% of income loss in Ghana. Concerted efforts by governments, international organisations and charities to combat the disease have resulted in a sharp decline in global malaria morbidity and mortality by 37 and 60% respectively between 2003 and 2015 [6]. However, nearly half of the world’s population, or 3.2 billion people, are still vulnerable to this disease. Sub-Saharan African regions, in particular, carry a disproportionately high share of the global malaria burden. In 2015, the region experienced approximately 88% of global malaria infections and 90% of malaria deaths [6].

Over the past decades, there has been a significant increase in investment towards the fight against malaria. WHO reports an increase in resources for malaria control from US$ 960 million in 2005 to US$ 2.5 billion in 2014 globally [6]. In Ghana, as part of an eight-year malaria strategic plan to bring down the incidence of malaria by 2015, the government pledged to contribute US$ 231 million in 2008. Donors, including The United States Agency for International Development (USAID), Presidents Malaria Initiative (PMI), The World Bank, United Nations Children’s Fund (UNICEF) and the private sector, all financially support malaria control programmes in Ghana [7]. PMI is an inter-agency initiative led by USAID. Launched in 2005, PMI has a task of reducing malaria-related mortality by 50% across 15 high-burden countries in sub-Saharan Africa including Ghana.

Unfortunately, the rising investment to control the disease does not lead to a significant fall in infection among young children. Contrary to the global trend, malaria infection rates among children under-five in Ghana have been rising despite the efforts and investments. As shown in Table 1, the number of reported cases of malaria among children under-five has been rising since 2000. Indeed, malaria accounts for more than half of Out Patient Department (OPD) cases of children under-five between 2010 and 2012, a sharp rise from 36.57% in 2000.

**Table 1:** Trends in reported malaria cases among children under-five from heath facilities in Ghana

	Malaria morbidity (OPD)			In-patient malaria cases			In-patient malaria death		
Year	< 5 OPD cases	< 5 malaria cases^a^	%	< 5 In- patient cases	< 5 in-patient malaria cases	%	< 5 Death	< 5 Malaria deaths	%
2000	1,411,860	516,337	36.57	98,507	27,478	27.89	8872	3952	44.54
2001	1,645,378	684,420	41.60	121,037	43,363	35.83	7804	2717	34.82
2002	1,184,624	518,081	43.73	133,963	42,887	32.01	8713	2914	33.44
2003	1,208,151	483,668	40.03	517,566	131,148	25.34	7636	2195	28.75
2004	1,485,451	513,449	34.57	844,091	196,429	23.27	5727	1380	24.10
2005	1,757,833	562,941	32.02	165,786	38,840	23.43	6610	2026	30.65
2006	1,772,727	579,947	32.71	52,429	10,602	20.22	3305	973	29.44
2007	3,417,098	1,056,331	30.91	113,952	22,019	19.32	5263	1241	23.58
2008	2,852,073	1,472,246	51.62	181,427	99,217	54.69	4901	1697	34.63
2009	3,635,219	1,003,612	27.61	250,796	122,575	48.87	6106	1505	24.65
2010	2,028,508	1,082,673	53.37	222,559	137,319	61.70	5345	1812	33.90
2011	3,130,270	1,709,549	54.61	343,085	129,110	37.63	5225	1539	29.45
2012	5,999,707	3,125,069	52.09	280,762	177,836	63.34	5044	1129	22.38

Source: Authors’ compilation using National Malaria Control Program (NMCP) routine data

^a^Malaria cases include clinical and confirmed cases of infection

Although in 2016 there was a significant reduction in the number of deaths attributable to malaria (1037 in 2015 to 590 in 2016) as well as the decline in Case Fatality Rate (CFR) (0.51 in 2015 to 0.32 in 2016) among children under-five, the proportion of cases attributable to malaria for under-five children remains high at 46.7% [8].

According to the Ghana National Malaria Control Programme (NMCP), malaria kills at least 3 children every day and also tops OPD cases in Ghana. While it is encouraging to observe a decline in in-patient deaths emanating from malaria infection among children under-five, the statistics on morbidity and in-patient cases remain alarming. This, therefore, raises questions about the role of malaria interventions and the type of effective strategies among children under-five in Ghana. In particular, the challenge is identifying the factors which play an important role in improving the protection offered by the malaria control tools. Utilising a sample of 2449 children in Ghana, we explore the impact of most popular intervention tools on malaria infection among children under-five.

We use an objective malaria infection measure that was not previously available in Ghana. In 2014, for the first time, children under-five were tested for malaria using a RDT in the GDHS. This objective measure makes it possible to test the association between IRS, ITN use and malaria infection in a large scale.

The rest of the paper is organized as follows. Section 2 gives an overview of malaria control tools in Ghana alongside with the literature review. The methodology and data are described in Section 3. Our main results are presented and discussed in Section 4, followed by robustness analysis in Section 5. The last section concludes with policy recommendations.

### Malaria control tools and the Ghanaian context

IRS and ITNs are the two main interventions recommended by the Global Malaria Programme in the WHO for malaria control. IRS is regarded as one of the most efficient vector control measures for tackling malaria transmission. It involves the spraying of walls and roofs of houses with long-acting chemical insecticides. This helps to kill the adult mosquitoes that dwell on such surfaces [9]. The role of IRS in reducing malaria infection is well-established with ample scientific evidence [10, 11]. Similarly, an empirical study on Ghana by Fuseini et al. [12] provide evidence on the efficacy of IRS.

In Ghana, IRS is carried out by the government, private individuals, and non-governmental organisations such as Anglogold Malaria (AGAMal), Global Fund and PMI. The use of IRS has been shown to have economic benefits in Ghana. For example, work absenteeism due to malaria infection has dropped from 6983 man-days in 2005 to 163 in 2010 due to the use of IRS [7]. However, due to the high cost of new insecticides for spraying, IRS exercise has reduced overtime. The PMI on IRS, for example, has scaled down its exercise from 9 to 4 districts in 2013 [7].

Similar to IRS, ITNs work as a vector control intervention to reduce malaria and other infections transmitted by insects [13–15]. Use of ITNs refers to hanging treated nets over one’s sleeping area to prevent mosquito bites [16]. Ghanaian government started to distribute ITNs to targeted groups through a multi prolonged distribution system in 1998. Beginning 2009, the Universal Coverage strategy was adopted and it aimed at providing one net to every two persons in a household through door-to-door distribution [7]. Distribution of bed-nets is also targeted at school pupils in order to increase its coverage. Pregnant women are also given ITNs on antenatal visits and all other individuals have the option of buying ITNs from shops/markets, pharmacy stores, and street vendors, among others [1]. Afoakwah et al. [17] have also shown that the use of ITNs can reduce under-five mortality by about 18.8% in Northern Ghana. Information on the direct impact of ITN use on malaria infection of the young children is however rare.

In addition to IRS and ITN use, BCC is also shown to be an important strategy through which malaria education is conveyed to communities, especially in malaria prone areas. In Ghana, malaria education in the form of prevention and treatments are conveyed through media sources including the television, radio, newspaper and magazines, posters, leaflets and brochures, and health workers and community volunteers [1]. The Ministry of Health (MoH) and the NMCP have adopted this approach to disseminate malaria information to Ghanaians in order to increase their knowledge and awareness, and to promote positive behavioural changes towards malaria prevention. Rhee et al. [18], for example, found that better knowledge levels about malaria significantly increased ITN use among households in Mali. Their controlled trial revealed that ITN use was greater among participants who had received malaria education compared to participants who did not receive any education. Ayi et al. [19] also found that malaria education improved school children’s perception about malaria in Ghana, and led to an increase in the proportion of adults using treated bed-nets. However, the effect of such efforts on malaria infection among children under-five in Ghana is still unclear. To this end, we include BCC strategies such as television, radio and print media (newspaper) in addition to IRS and ITN to evaluate their association with malaria infection rates among children under-five.

## Methods

Building on the current literature, this study uses a logit model to estimate the likelihood that a child gets infected with malaria. The model considers child’s attributes and household effects as well as observed malaria control measures including ITN, IRS and BCC. The aim is to control for covariates that theoretically and intuitively impact the child’s propensity to being infected. The corresponding logit model is specified as:

1 $$ M={\alpha}_i+\beta {I}_i+\pi {E}_i+\eta {MP}_i+{u}_i\  with\ M=\left\{\underset{0\kern0.5em otherwise,}{\overset{1\kern1em if\kern0.5em {M}^{\ast }>0}{}}\right. $$

where *M*^∗^ is a latent variable that is unobserved. *M* is a dummy variable that takes the value of 1 if the test outcome is positive and 0 if negative; *I* is a vector of variables for individual child attributes such as age, weight and gender. *E*captures environmental factors such as parental education, rural dwelling, household size and household wealth. *MP* captures malaria preventive measures, ITN use, IRS, and BCC. *u* is the error term.

IRS is a dummy variable with the value 1 if the child slept in a room that has been sprayed in the last 6 months. ITN, as captured by the GDHS report, includes factory-treated net that does not need further treatment, or a pre-treated net obtained within the past twelve months, or a soaked net with insecticides within the past twelve months. There are two indicators of ITN use in the data set: mother’s use of ITN and child’s use of ITN. It is common practice in Africa that mothers sleep with their young children [20]. Therefore the mother and child’s use of ITN is a better measure of the use of ITN as a preventive tool for young children. The data indicated that malaria infection is higher among children who slept under ITN alone than those who did not (49.77% vs 36.89%). A possible explanation is that children are more likely to be put under ITN after they show symptoms of fever or tested positive for malaria. In other words, ITN is used as a treatment method instead of a preventive tool. This could also be due to social desirability on the part of the parents in reporting use of ITNs by their children. Given the fact that it is popular in Ghana for mothers to sleep with their young children, we consider ITN use only when both mother and child use it.^1^

Regarding behavioural change communication, we include media source of malaria education such as audio (radio), audio-visual (television), and print media (newspaper), mainly due to their high utilisation rate among the population. Other sources were not included because this was not a mutually exclusive response, possibly causing multicollinearity. In estimating Eq. (1), the Maximum Likelihood Estimation (MLE) technique (specifically a logit model that has a flatter tail compared to a probit model) is employed. The logit model is appropriate as the study seeks to provide information on the odds of being infected with malaria.

The study uses secondary data sourced from the current round of the Ghana Demography and Health Survey [1]. This survey is conducted every five years since 1988, with 2014 being the most recent wave, and it is collected from rural and urban areas in all ten regions in Ghana. These data sets are collected by the Ghana Statistical Service (GSS) and the Ghana Health Service (GHS) with support from the National Public Health Reference Laboratory (NPHRL) of the GHS. During the 2014 survey, field health assistants collected capillary blood samples from children aged 6 to 59 months in half of the households surveyed. The health assistants then tested for malaria on the field using a RDT with consent from the parent or the guardian. The outcome of the test was then recorded as either positive or negative. Children with positive results are considered as having malaria infection. About 2698 children had their blood samples tested, which represents 97% of all eligible children [1]. Demographic information is also collected via parental self-reports.

The sample used for this study comprises 2449 children aged between 6 and 59 months in Ghana. This represents 90.77% of the total children who were involved in the malaria test exercise. The remaining 9.23% were excluded due to missing information on some of the covariates used for the regression analysis. Descriptive statistics are presented in Table 2. 40.50% of children have tested positive. The average age is 32 months and 52% are boys, with an average weight around 12 kg. With regards to household attributes, average family size was 6 persons and 60.20% lived in rural areas. A significant proportion (73.20%) lived in poor households. Household wealth index used in this study was constructed by the GSS using Principal Components Analysis (PCA). Items such as television, bicycle or car, as well as dwelling characteristics, such as a source of drinking water, sanitation facilities and type of flooring material are used to compute this index (see [1] for further details). It is noteworthy that 57.10% of mothers have no secondary education and 32.40% of mothers have no formal education at all. Fathers have significantly higher levels of education compared to mothers.

**Table 2:** Summary statistics of variables (*N* = 2449)

	Variable Definition	Mean	SD
Malaria	Whether the child tested positive for malaria (1/0)	0.405	0.491
Age	Child age in months	31.653	15.350
Female	Whether the child is female (1/0)	0.481	0.499
Child weight	Child weight in kilograms	11.976	3.019
Rural	Whether the child lives in a rural area (1/0)	0.602	0.489
Household size	Total household members	5.867	2.737
Poorest	Whether the child belongs to a poorest household (1/0)	0.324	0.468
Poorer	Whether the child belongs to a poorer household (1/0)	0.219	0.414
Middle	Whether the child belongs to a middle income household (1/0)	0.189	0.391
Richer	Whether the child belongs to a richer household (1/0)	0.149	0.356
Richest	Whether the child belongs to a richest household (1/0)	0.120	0.324
Mother’s education			
No education	Whether the mother has no level of education (1/0)	0.367	0.482
Primary	Whether the mother has completed primary education (1/0)	0.204	0.403
Secondary and above	Whether the mother has completed at least secondary education (1/0)	0.429	0.495
Father’s education			
No education	Whether the father has no level of education (1/0)	0.316	0.465
Primary	Whether the father has completed primary education (1/0)	0.193	0.395
Secondary and above	Whether the father has completed at least secondary education (1/0)	0.490	0.500
Not protected	Whether the child uses no preventive tool	0.382	0.486
ITN	Whether the mother and child used ITN the night before the survey (1/0)	0.402	0.490
IRS	Whether the child slept in indoor residual sprayed room during the past twelve months (1/0)	0.132	0.338
ITN and IRS	Whether the child use ITN and also sleeps in indoor residual sprayed room	0.084	0.278
Television	Whether the household received malaria education via television (1/0)	0.520	0.500
Radio	Whether the household received malaria education via radio (1/0)	0.784	0.411
Newspaper	Whether the household received malaria education via newspapers (1/0)	0.091	0.288

Source: Author’s own computation from GDHS 2014 data

38.2% of children in our sample were not protected by either ITN or IRS, and 40.20% of children and their mothers slept under ITN the night before the survey and only 13.20% of children slept in a room that has been sprayed. The remaining 8.40% were covered by both ITN and IRS. In terms of malaria education, it was found that most of these households (78.4%) receive malaria education via radio, followed by television (52%) and newspaper (9.10%).

Table 3 presents the prevalence of malaria among children under-five for the entire sample, and for various sub-groups. There is not much difference between boys and girls in terms of infection rate, but the prevalence of malaria among rural children (56.08%) is more than twice of that among urban children (21.71%). Maternal education level is found to be highly correlated with malaria infection among children. Children whose mother has at least secondary education are the least vulnerable groups while children whose mother has no education record the highest prevalence of malaria infection. Relative to ITN use, children who sleep in rooms with IRS are less vulnerable to malaria infection. Children whose carers receive malaria education through television have lower rates of malaria infection than those reporting to have received education through radio or print media (newspaper).

**Table 3:** Dynamics of malaria prevalence among children under five (*N* = 2449)

Variables	Malaria prevalence (%)
Male children	42.80
Female children	41.52
Children in rural areas	56.08
Children in urban areas	21.71
Mothers with no education	54.20
Mothers with primary education	47.31
Mothers with secondary education and above	25.80
Children who slept in indoor residual sprayed room	36.50
Children who slept under ITN	44.97
Malaria education through radio	40.44
Malaria education through television	28.93
Malaria education through newspapers	29.55

Source: Author’s own computation from GDHS 2014 data

## Results

In order to observe how individual attributes, environmental factors and malaria preventive measures interact with risk of malaria infection, a step-wise regression is employed. The results are reported in Table 4. The Chi-square statistics (a goodness-of-fit test) suggest that all four specifications present regression lines that are a good fit at 1 % significance level. In the first specification, the impact of individual characteristics of the children is tested. Age and weight are the only significant variables, and gender does not play any significant role in malaria infection. We then add the environmental factors and present our results in specification 2. We observe a decrease in the coefficient and the odds ratio for age, but an increase for weight. Moreover, most of the environmental variables are also statistically significant, except for the poorer households when compared the poorest ones, and mother’s primary education when compared to no education. Specification 3 then adds malaria preventive measures to specification 2, followed by addition of interaction variables in the last specification. In specifications 2 to 4, the coefficients and the odds of malaria infection are very similar for the age and weight variables. Weight also has a non-linear effect on the odds of malaria infection. Results for the weight and weight-squared variables suggest an increase in the odds of malaria infection among children weighing up to 11.5 kg but a reduction in the odds for those above this weight limit. The reflection point is computed using the coefficients for the weight and weight-squared variables in Table 4. Similar to the child attributes, we observe little variation in the coefficient and the odds ratios for the environmental factors when specification 2 results are compared to the ones in specifications 3 and 4. As a result, we focus our discussion on the results for the individual, environmental, and malaria control variables from specifications 3 and 4.

**Table 4:** Logit regressions for determinants of malaria infection among children under-five

	Spec. 1		Spec. 2		Spec. 3		Spec. 4	
	I	Odds ratio	I + E	Odds ratio	I + E + MP	Odds ratio	I + E + MP+(MP^a^E)	Odds ratio
Individual attributes (I)								
Age	**0.040**^**c**^ (0.005)	**1.041**	**0.023**^**c**^ (0.006)	**1.023**	**0.022**^**c**^ (0.06)	**1.023**	**0.024**^**c**^ (0.006)	**1.023**
Female	−0.080 (0.085)		−0.047 (− 0.094)		− 0.057 (0.097)		− 0.040 (0.096)	
Child weight	**0.241**^**b**^ (0.109)	**1.272**	**0.454**^**c**^ (0.118)	**1.574**	**0.432**^**c**^ (0.122)	**1.540**	**0.447**^**c**^ (0.128)	**1.563**
Weight_sq	**−0.016** ^**c**^ **(0.004)**	**0.985**	**−0.020**^**c**^ (0.004)	**0.981**	**−0.019**^**c**^ (0.005)	**0.981**	**−0.019**^**c**^ (0.005)	**0.981**
Environmental factors (E)								
Rural			**0.579**^**c**^ (0.124)	**1.785**	**0.591**^**c**^ (0.129)	**1.805**	**0.557**^**c**^ (0.129)	**1.745**
Hhsize			**0.070**^**c**^ (0.019)	**1.072**	**0.063**^**c**^ (0.020)	**1.065**	**0.071**^**c**^ (0.019)	**1.074**
Wealth index (Base = Poorest)								
Poorer			0.116 (0.124)		−0.093 (0.134)		−0.089 (0.131)	
Middle			**−0.446**^**c**^ (0.146)	**0.640**	**−0.636**^**c**^ (0.164)	**0.529**	**−0.441**^**c**^ (0.158)	**0.643**
Richer			**−1.507**^**c**^ (0.205)	**0.222**	**−1.647**^**c**^ (0.235)	**0.193**	**−1.422**^**c**^ (0.225)	**0.241**
Richest			**−1.965**^**c**^ (0.277)	**0.140**	**−2.017**^**c**^ (0.290)	**0.133**	**−1.807**^**c**^ (0.297)	**0.164**
Mother’s education (Base = no education)								
Primary			0.166 (0.145)		0.085 (0.151)		−0.146 (0.188)	
Secondary and above			**−0.557**^**c**^ (0.135)	**0.573**	**−0.688**^**c**^ (0.145)	**0.503**	**−0.347**^**b**^ (0.172)	**0.749**
Father’s education (Base = no education)								
Primary			−0.211 (0.195)		**−0.312**^**a**^ (0.168)	**0.732**	**−0.352**^**b**^ (0.162)	**0.693**
Secondary and above			**−0.483**^**c**^ (0.141)	**0.617**	**−0.511**^**c**^ (0.151)	**0.600**	**−0.352**^**c**^ (0.149)	**0.677**
(Base = not protected)								
ITN					−0.001 (0.111)			
IRS					−1.167^c^ (0.169)	**0.312**	**−0.916**^**c**^ (0.151)	**0.400**
Both ITN and IRS					−1.113^c^ (0.194)	**0.329**		
BCC strategies								
Television					**−0.325**^**c**^ (0.115)	**0.722**	**−0.336**^**c**^ (0.115)	**0.715**
Radio					0.009 (0.118)		−0.010 (0.116)	
Newspaper					−0.216 (0.186)			
Interactions								
ITN^a^primary education							0.409^a^ (0.213)	
ITN^a^secondary education							**−0.289**^**c**^ (0.148)	**0.545**
Newspaper^a^primary education							0.501 (0.374)	
Newspaper^a^secondary education							**−1.432**^**c**^ (0.517)	**0.239**
Constant	−0.829^c^ (0.630)		−4.343^c^ (0.770)		−3.567^c^ (0.804)		−3.965^c^ (0.786)	
Reflection point for child weight	**7.43**		**11.56**		**11.49**		**11.48**	
Pseudo R2	0.0251		0.175		0.206		0.197	
Prob > chi2	86.28^c^		404.86^c^		449.34^c^		442.60^c^	
*N*	2462		2462		2449		2449	

Source: Author’s own computation from GDHS 2014

Robust standard errors are in parentheses

^a^, ^b^, ^c^ Significant at 10, 5 and 1%, respectively

Bolded variables are statistically significant

## Discussion

### Individual attributes

A child’s odds of being infected by malaria are positively associated with his/her age. This relationship may not be linear and the non-linearity may be explained by weight and weight-squared. Given that the turning point for weight (11.5 kg) corresponds to the mean weight, it suggests that children become more resistant to diseases after reaching a certain weight. This could be an indicator of an improved immune system after 37 months of age as shown in Table 5 of the sensitivity analysis. Also at the age the child weights 11.5 kg, he/she may sleep with his/her mother under one ITN and would receive a better protection from the net. The gender dummy variable is not significant in all four regressions. This finding seems to be different from the experimental observation by Cernetich et al. [21] where they noticed that females had faster recovery from anaemia, induced weight loss and had reduced mortality.

### Environmental factors

Due to the endemic nature of malaria in Ghana, we investigate the influence of the environment surrounding the children. We control for rural dwelling, household size, household wealth and parental education. Each of these contextual factors significantly influences the odds of malaria infection among children under-five. The prevalence of malaria in rural areas is reported to be about 38% compared to 15% for people who live in urban environments [1]. Consistent with the GDHS [1] our study shows that living in a rural area increases a child’s odds of being infected. The dichotomous rural dwelling variable is statistically significant in both specifications at 1 % level. Children in rural areas are about 75 to 80% more likely to be infected with malaria than their counterparts in urban settings. This could potentially be due to the low use of intervention tools among rural folks. For example, the GDHS [1] reports that while 64% of rural dwellers have access to ITN, only 47% use the net. Another reason is that most in rural areas are wetlands used for farming activities [22] which present breeding places for mosquitoes. This finding emphasizes the need for critical attention to children in rural areas.

With regards to parental education, we show that higher parental education is associated with lower odds of malaria infection among their children. Secondary education of parents has the highest significant effect on malaria prevention. Maternal education is also argued to play a significant role in child health outcomes through increased labour market earnings [23] and changes in individual behaviour through increased autonomy [24].

Similarly, children who live in large households are more likely to have malaria infection. Having one additional member in the household increases the likelihood of infection by about 6.5%. With a mean household size of 5.89 (Standard deviation 2.73), a plausible explanation is that the presence of congestion in homes may produce better breeding grounds for mosquitoes and less time and resources for preventive measures. In addition, a larger household means less space for sleeping and this makes it difficult to mount ITNs to protect all household members. It is common in rural Ghana that an area is used for living room in the day time and a bedroom at night. In such cases, the ITN needs to be mounted on the wall every night. This is a burden for the household and may discourage the use of an ITN. Justeson and Kunst [25] suggest that the risk of disease spread among groups of persons living together is significantly correlated with the level of nucleation that exists among them. Large households might also tend to be busy and early disease symptoms, especially in young children, can remain unnoticed, resulting in delays in treatment and therefore lead to more adverse effects [26].

We also show that malaria infection is highly correlated with household wealth. The odds of malaria infection among children aged below five years decreases as household wealth rises. Compared to the poorest households, children from richer households are about 77.8% less likely to be infected with malaria, and the odds are 86% lower for children in the richest households, all else being equal. This may be attributed to the fact that wealthier families can afford better goods and services leading to more positive health outcomes. Arthur [27] found a similar correlation between wealth and antenatal care in Ghana. It is therefore important to note that poorer households should be prioritized in any mass distribution of ITNs as well as IRS campaigns. This form of prioritisation is critical especially since the summary statistics suggest that 54.3% of these children live in poorer households.

### Malaria control measures

As mentioned earlier, in Ghana ITNs have been freely distributed to households by public and private organisations since 1998 [7]. The GDHS report shows that about 85% of ITNs were obtained in this way. IRS is only implemented by government and non-governmental organisations in selected districts of Ghana depending on financial costs and technical feasibility [1]. Therefore, a significant proportion of households not covered by IRS had to rely on ITN as the main preventive measure.

Of the two main preventive measures, we find no significant association between ITN use and malaria infection among young children. Previous studies identified two main reasons that may undermine the protection offered by ITN use. First, the ITNs used may have lost their insecticidal protection, making the nets less efficient [28]. Second, some users may not lay out the net correctly due to the perceived heat increase and fear of suffocation that come with the use of ITNs [29, 30].

IRS, on the other hand, is associated with reduced incidence of malaria infection. Children who sleep in rooms that have been sprayed in the last 6 months are about 68% less likely to be infected with malaria than children who do not, all else being equal. This finding supports similar evidence presented by Loha et al. [10] that IRS significantly reduces the incidence of falciparum malaria. The dual use of both ITN and IRS, however, does not provide an added protection.

Although ITN use alone is not significantly linked with a reduction of malaria infection, specification 4 suggests that ITNs used by educated mothers are associated with a lower incidence of malaria. We interact only mother’s education because they are the primary care giver of the child. For children whose mother has at least secondary education, use of ITN reduces their odds of malaria infection by 45.50%. Inasmuch as this finding gives credence to Gary Becker’s human capital theory, the implication here is that educated mothers are more likely to be aware of the benefits of using ITN and are capable of the appropriate use of the net to protect their children. Our finding is consistent with findings that in Botswana and Zimbabwe educated mothers were more successful in reducing the prevalence of diarrhoeal diseases among the children [31].

With regards to behavioural communication change measures, our study analyses how three forms of media – television, radio and newspaper impacts malaria infection among children. Although a significant percentage of households receive malaria education through the radio (78.4%), no significant association has been identified with radio exposure and malaria infection reduction. Malaria education via television is the only variable with an independent significant link with child’s malaria infection. On average, malaria education through television reduces the odds of infection by 26.9%, keeping all else constant.

While malaria education through print media did not show a significant independent effect, it was significant for children whose mother had at least secondary education. This is not surprising because it is more likely that educated parents can read, understand and communicate information conveyed through print media. These findings suggest that television and print media play a critical role in disseminating information on malaria but providers need to be aware of the audience. Given that nearly 60% of carers were educated at primary level or below, audio-visual (television) forms of malaria education appear to be the most effective method.

Our study has shown that the effect of the IRS is far more than of that for ITN use. We have also demonstrated the important role of mother’s education in facilitating the protection provided by malaria intervention tools, especially ITN use. Thus, our results echo the crucial need to educate the users before implementing an intervention that requires correct use. We also find that an association between mother’s exposure to print media and children’s infection rate, but only if the mother has completed at least secondary education. However, a more effective way of communicating behaviour change towards malaria control is the television.

## Sensitivity analysis

It is probable for the effectiveness of the malaria preventive measures as well as some of the explanatory variables to differ depending on the age of the child or the wealth of the household. Thus, as a sensitivity analysis, we run the regressions for various age cohorts and household wealth groups. We run the regressions for both specifications 3 and 4, and report only the odds ratios. For comparison reasons, the main results for specification 3 are presented at the top panel, and the bottom section includes all the variables from specification 4.

For the age-cohort regressions, presented in Table 5, we divide the sample into three; 6 to 24 months, 25 to 36 months, and 37 to 59 months. Focusing on the results for specification 3, we show that ITN does not have a significant effect on malaria infection in none of the age cohorts. Although the coefficient for the ITN variable is not significant, it has a negative effect on malaria among children up to two years old, which supports the assertion that younger children sleep with their mothers and are protected through proper use. However, IRS has a sizable effect on the odds ratios for all age cohorts. The results for using both ITN and IRS does not differ much compared to the only IRS odds ratios. Although the dual use of both ITN and IRS does not provide an added advantage to the youngest cohort, our results show that for children above 2 years of age, a combination of the two offers better protection.

Child weight has a notable effect on incidence of malaria infection depending on the age cohort. For young children and babies child weight increases the incidence of malaria infection, while we observe the opposite effect for older children aged 37 to 59 months. Weight gain among children aged 37 months and above corresponds to a decline in the odds of malaria infection. Living in a rural area and living in a more crowded household cause an increase in malaria infection rates. The only exception is for the effect of household size on older children; we find no statistical significance. Household wealth has the expected effect on malaria infection and the results are consistent among age cohorts. Richer and the richest households significantly lower the malaria infection incidence irrespective of the child’s age. An interesting finding is the effect of the education variables. More educated parents significantly lower the malaria infection incidence for only the oldest cohort.

Regarding the malaria prevention measures, the IRS variable has a significant effect in lowering malaria incidence, and this effect is slightly lower in specification 4 compared to specification 3 for all age cohorts. ITN and the mother’s education interaction terms are all insignificant except for the 2–3 year old children. Having a more educated mother significantly lowers the malaria incidence for these households. Finally, among the BCC strategies, we observe that television has a significant effect except for the oldest cohort, and the effectiveness of the newspaper depends on the mother’s education level.

**Table 5:** Age-cohort regression for determinants of malaria infection among children under-five

	6–24 months Odds ratio	25–36 months Odds ratio	37–59 months Odds ratio
ITN (Specification 3)	0.932 (−0.37)	1.094 (0.39)	1.062 (0.34)
IRS (Specification 3)	**0.313**^**c**^ (−4.24)	**0.419**^**b**^ (−2.41)	**0.279**^**c**^ (− 4.66)
Both ITN and IRS (Specification 3)	**0.402**^**c**^ (− 2.93)	**0.291**^**c**^ (− 2.75)	**0.263**^**c**^ (− 4.42)
Individual attributes (I)			
Female	0.963 (−0.23)	1.037 (0.18)	0.982 (−0.13)
Child weight	**1.213**^**c**^ (4.01)	1.025 (0.38)	**0.909**^**b**^ (−2.53)
Environmental factors (E)			
Rural	**1.539**^**b**^ (2.04)	**1.925**^**b**^ (2.50)	**1.896**^**c**^ (3.21)
Hhsize	**1.056**^**b**^ (1.99)	**1.180**^**c**^ (3.47)	1.047 (1.43)
Wealth index (Base = Poorest)			
Poorer	1.221 (0.93)	1.217 (0.70)	0.924 (−0.37)
Middle	0.721 (−1.27)	0.869 (− 0.41)	**0.483**^**c**^ (−2.85)
Richer	**0.293**^**c**^ (−3.19)	**0.193**^**c**^ (−3.23)	**0.214**^**c**^ (−4.55)
Richest	**0.041**^**c**^ (−4.10)	**0.272**^**b**^ (− 2.4)	**0.188**^**c**^ (− 3.72)
Mother’s education (Base = no education)			
Primary	0.856 (−0.59)	0.821 (− 0.57)	0.739 (−1.07)
Secondary and above	0.768 (−1.07)	0.931 (−0.24)	**0.611**^**b**^ (−2.05)
Father’s education (Base = no education)			
Primary	0.724 (−1.23)	0.799 (−0.64)	**0.639**^**a**^ (− 1.69)
Secondary and above	**0.666** ^**a**^ **(−1.66)**	0.876 (− 0.37)	**0.605**^**b**^ (− 2.03)
Malaria preventives measures (MP)			
Protection (Base = Not protected)			
IRS alone	**0.389**^**c**^ (−3.88)	**0.486**^**b**^ (− 2.20)	**0.374**^**c**^ (−4.13)
ITN^a^prim_edu	1.115 (0.31)	2.501^a^ (1.84)	1.646 (1.49)
ITN^a^sec_edu	0.621 (−0.94)	**0.291**^**b**^ (−2.20)	0.634 (−1.10)
BCC strategies			
TV	**0.692**^**a**^ (−1.88)	**0.505**^**c**^ (−2.86)	0.869 (−0.78)
Radio	1.134 (0.67)	0.779 (−1.04)	1.013 (0.07)
Newspaper^a^prim_edu	**5.240**^**b**^ (2.54)	**0.085**^**c**^ (−2.79)	2.482 (1.56)
Newspaper^a^sec_edu	**0.09**^**c**^ (−2.73)	5.752 (1.51)	**0.129**^**b**^ (−2.57)
Constant	0.066^c^ (−2.73)	0.185 (−1.59)	2.361 (1.09)
Pseudo R2	0.179	0.238	0.195
Prob > chi2	130.15^c^	121.88^c^	186.83^c^
*N*	906	563	991

Source: Author’s own computation from GDHS 2014 data

T-statistics are in parentheses

^a^, ^b^, ^c^ Significant at 10, 5 and 1%, respectively

Bolded variables are statistically significant

To analyse our findings by varying household wealth, in Table 6, we present the results after dividing the sample into three categories; poor, middle-class, and rich. An interesting observation from specification 3 results is that there is a statistically significant link between IRS and malaria infection of young children among poor and middle-class households, but such a link is absent among the rich households. Moreover, the effect of using both ITN and IRS is significant only for the poor households.

**Table 6:** Logit regressions based on household wealth index (Model 4)

	Poor^a^ Odds ratio	Middle class^a^ Odds ratio	Rich^a^ Odds ratio
ITN (Specification 3)	0.805 (− 1.41)	1.325 (1.28)	1.229 (0.71)
IRS (Specification 3)	**0.249** ^**d**^ **(−7.35)**	**0.363** ^**b**^ **(−1.88)**	1.112 (0.20)
Both ITN and IRS (Specification 3)	**0.257** ^**d**^ **(−6.43)**	0.752 (−0.43)	1.193 (0.24)
Individual attributes (I)			
Age	**1.019**^**d**^ (2.61)	**1.038**^**c**^ (2.56)	**1.029**^**b**^ (1.68)
Female	0.944 (−0.48)	1.095 (0.43)	0.779 (−0.90)
Child weight	**1.575**^**d**^ (3.16)	**1.672**^**b**^ (1.66)	1.597 (1.14)
Weight square	**0.982**^**d**^ (−3.43)	**0.974**^**c**^ (−2.21)	0.977 (−1.34)
Environmental factors (E)			
Rural	**1.807**^**d**^ (3.28)	**1.529**^**b**^ (1.91)	**2.071**^**c**^ (2.03)
Hhsize	**1.048**^**c**^ (02.15)	**1.232**^**d**^ (4.13)	1.046 (0.55)
Mother’s education (Base = no education)			
Primary	**0.679**^**b**^ (−1.91)	1.350 (0.75)	2.697 (1.55)
Secondary and above	0.605^c^ (−2.15)	1.11 (0.35)	1.850 (1.07)
Father’s education (Base = no education)			
Primary	**0.651**^**c**^ (−2.37)	0.878 (−0.24)	1.356 (0.32)
Secondary and above	**0.618**^**d**^ (−2.81)	0.888 (−0.25)	0.647 (− 0.53)
Malaria preventive measure (MP)			
IRS	**0.372**^**d**^ (−6.12)	**0.311**^**c**^ (−2.23)	1.056 (0.10)
ITN^b^prim_edu	1.438 (1.42)	1.300 (0.58)	**3.544** ^**c**^ **(2.57)**
ITN^b^sec_edu	0.699 (−1.02)	0.711 (−0.63)	**0.123** ^**d**^ **(−3.23)**
BCC strategies			
TV	**0.795**^**b**^ (−1.66)	**0.611**^**c**^ (−2.09)	0.712 (−0.71)
Radio	0.989 (−0.08)	0.992 (−0.03)	1.109 (0.24)
Newspaper^b^prim_edu	0.194 (0.37)	1.862 (0.391)	1.069 (0.11)
Newspaper_sec_edu	**0.181**^**c**^ (−2.28)	0.314 (−1.08)	0.552 (−0.75)
Constant	0.218^d^ (−4.02)	**0.004**^**c**^ (−2.87)	**0.002**^**c**^ (−2.31)
Pseudo R2	0.079	0.091	0.097
Prob > chi2	121.31^d^	50.83^d^	43.69^d^
*N*	1335	463	664

Source: Author’s own computation from GDHS 2014 data

T-statistics are in parentheses

^a^Poorest and poorer = poor, middle = middle class, richer and richest = rich

^b^, ^c^, ^d^ Significant at 10, 5 and 1%, respectively

Bolded variables are statistically significant

Similar to our previous findings, child weight has a positive effect on malaria incidence while the weight square has a negative effect. Both of these variables, however, are not significant for the rich households. Living in a rural area and in a crowded household again increases the odds of malaria infection except for the effect of household size on children in richer families. Parent’s education matters for the poor households and lowers the malaria incidence rates for children. However, a similar effect of parent’s education on malaria is missing for the middle-class and rich households.

Comparable to our finding in specification 3, the IRS is significant only for the poor and the middle-class households. However, the ITN variable interacted with mother’s education is only significant for the rich households. Similar to IRS, Television as a BCC strategy is only significant for the poor and the middle-class households. Another BCC strategy, newspaper, has a significant effect in lowering malaria incidence when interacted with mother’s education and this holds only for the poor households.

Overall, we find that our explanatory variables and malaria preventive measures have more of a significant effect on the poor households. The significance of our findings diminishes as the household wealth increases, with the least significant effects for the richer households. This is of great importance for the government while identifying the households to target their policies towards. Our findings also have crucial implications in terms of policy recommendations which we discuss extensively in the next section.

## Conclusion

This paper explores factors influencing malaria infection among children under-five from three dimensions: individual attributes, living environment and intervention measures. Three conclusions can be drawn. First, we identify vulnerable groups among children under-five. Our results show that children living in a large family with low income in rural areas are most prone to malaria infection. In addition, our results suggest that children under age three are more prone to malaria infection. Therefore, children must be given critical attention during their early formative years until their immune systems have significantly improved. Second, this study has shown that the most effective tool to prevent malaria infection among children under-five is IRS and its protection far exceeds that noted for ITN use. Given that the number of young children protected by IRS is half that of those protected by ITN, policy makers should consider increasing the use of IRS. Such a policy change promises more significant positive results than trying to increase ITN use. This is because, unlike ITN, the effect of IRS is not moderated by any human capital attribute. In a developing country like Ghana, where educational levels are very low especially among women, intensifying IRS is the most efficient way of preventing malaria among children in poor and rural households. Third, mother’s education moderates the protection of ITN use and BCC strategy through print media. Mother’s education was found to be an important ingredient in the campaign to reduce malaria infections. Education enhances the role of ITN use and also strengthens BCC campaigns aiming at improving the efficacy of malaria preventive and curative measures.

Drawing on the findings of this study, we recommend that policy makers implement an intervention program tailored for various social and economic groups. First, prioritise the use of IRS to poorer and rural households where primary carers have low levels of education. Due to the significant cost associated with IRS, resources should be directed to assist the poorest households and communities where the use of ITN is less likely to be effective. Second, distribution of ITNs should be accompanied with a multi-media education program to enhance its protection. Television education offers the best protection compared to other forms. However, given its limitation in providing detailed information, other types of media should also be utilised. Care needs to be taken to ensure that families and carers who are less literate can be assisted to understand the information provided. Thirdly, more attention must be paid to educate mothers and mothers-to-be. The significant impact of an educated mother on the health outcomes of children suggests educating women not only benefits them by enhancing their human capital, but also benefits the family and community with improved child health. More effort and resources should be diverted to lift the education level of women in Ghana to benefit the nation as a whole. Inasmuch as this study does not observe a significant independent effect of ITN use on malaria infection, we recommend that future surveys consider continuous use of the ITN tool in order to ascertain its independent effect on malaria among this vulnerable group.

Although our study has outlined some policy implications, it is limited by the cross sectional nature of our data. Thus, our data makes it difficult to evaluate the efficacy of the intervention tools. Our findings demonstrate the associations rather than confirm effectiveness. Another limitation is associated with the measurement for ITN use. Since the surveyed households were only asked whether the child slept under ITN the night before, our ITN measure does not necessarily mean that the child had been protected by ITN on continuous basis. We would suggest future surveys to include a question on the frequency of the use of ITN in a longer period of time. Finally, the analysis is limited by our inability to control for some cofounders such as areas in Ghana that could have high malaria endemic. However, we believe that any bias coming from such exclusion is mitigated by the inclusion of rural dummy variable since such areas are more likely to be in rural areas.

## Abbreviations

AGAMal: Anglogold Malaria
BCC: Behaviour change communication
GDHS: Ghana demographic and health surveys
GHS: Ghana health service
GSS: Ghana statistical service
IRS: Indoor residual spraying
ITN: Insecticide treated bed-nets
MoH: Ministry of health
NMCP: National Malaria control programme
NPHRL: National public health reference laboratory
OPD: Out Patient Department
OR: Odds ratio
PCA: Principal components analysis
PMI: Presidents Malaria initiative
RDT: Rapid diagnostic test
UNICEF: United Nations children’s fund
USAID: United States Agency for International Development
WHO: World Health Organization

## Footnotes

1 Preliminary regressions suggested that mother and child use of ITN better explained malaria infection rate compared to when the child alone uses the net. This is due to malaria incidence for children sleeping under ITN alone, as mentioned earlier. The results on ITN use by child alone is available upon request.
